# Lignocellulose-Adapted Endo-Cellulase Producing Streptomyces Strains for Bioconversion of Cellulose-Based Materials

**DOI:** 10.3389/fmicb.2016.02061

**Published:** 2016-12-22

**Authors:** Valeria Ventorino, Elena Ionata, Leila Birolo, Salvatore Montella, Loredana Marcolongo, Addolorata de Chiaro, Francesco Espresso, Vincenza Faraco, Olimpia Pepe

**Affiliations:** ^1^Department of Agricultural Sciences, University of Naples Federico IINaples, Italy; ^2^Institute of Biosciences and BioResources—National Research CouncilNaples, Italy; ^3^Department of Chemical Sciences, University of Naples Federico IINaples, Italy

**Keywords:** *Streptomyces* spp., endo-cellulase, *Arundo donax*, saccharification, biochemicals and bioenergy

## Abstract

Twenty-four *Actinobacteria* strains, isolated from *Arundo donax, Eucalyptus camaldulensis* and *Populus nigra* biomass during natural biodegradation and with potential enzymatic activities specific for the degradation of lignocellulosic materials, were identified by a polyphasic approach. All strains belonged to the genus *Streptomyces* (*S*.) and in particular, the most highly represented species was *Streptomyces argenteolus* representing 50% of strains, while 8 strains were identified as *Streptomyces flavogriseus* (synonym *S. flavovirens*) and *Streptomyces fimicarius* (synonyms *Streptomyces acrimycini, Streptomyces baarnensis, Streptomyces caviscabies*, and *Streptomyces flavofuscus*), and the other four strains belonged to the species *Streptomyces drozdowiczii, Streptomyces rubrogriseus, Streptomyces albolongus*, and *Streptomyces ambofaciens*. Moreover, all *Streptomyces* strains, tested for endo and exo-cellulase, cellobiase, xylanase, pectinase, ligninase, peroxidase, and laccase activities using qualitative and semi-quantitative methods on solid growth medium, exhibited multiple enzymatic activities (from three to six). The 24 strains were further screened for endo-cellulase activity in liquid growth medium and the four best endo-cellulase producers (*S. argenteolus* AE58P, *S. argenteolus* AE710A, *S. argenteolus* AE82P, and *S. argenteolus* AP51A) were subjected to partial characterization and their enzymatic crude extracts adopted to perform saccharification experiments on *A. donax* pretreated biomass. The degree of cellulose and xylan hydrolysis was evaluated by determining the kinetics of glucose and xylose release during 72 h incubation at 50°C from the pretreated biomass in the presence of cellulose degrading enzymes (cellulase and β-glucosidase) and xylan related activities (xylanase and β-xylosidase). The experiments were carried out utilizing the endo-cellulase activities from the selected *S. argenteolus* strains supplemented with commercial β-gucosidase and xylanase preparations from Genencore (Accellerase BG and Accellerase XY). Cellulose and xylan conversion, when conducted using commercial (hemi)cellulases, gave glucose and xylose yields of 30.17 and 68.9%, respectively. The replacement of the cellulolytic preparation from Genencor (Accellerase 1500), with the endo-cellulase from *S. argenteolus* AE58P resulted in almost 76% of the glucose yield obtained in the presence of the commercial counterpart. Due to the promising results obtained by using the enzymatic crude extracts from *S. argenteolus* AE58P in the pretreated *A. donax* saccharification experiments, the proteins putatively responsible for endo-cellulase activity in this strain were identified by proteomics. Several proteins were confidently identified in different *Streptomyces* spp., eight of which belong to the class of Carbohydrate active enzymes. Overall results highlighted the biotechnological potential of *S. argenteolus* AE58P being an interesting candidate biocatalyst-producing bacterium for lignocellulose conversion and production of biochemicals and bioenergy.

## Introduction

Cellulose is the most abundant renewable carbohydrate source on Earth and its bioconversion into second-generation biofuels or other high-added-value products is emerging as the most promising alternative technology to overcome the issues associated with utilization of fossil carbon sources (Haberl et al., 2010). In this context, dedicated energy crops, such as miscanthus, giant reed, poplar, switchgrass, reed canary grass, willow, and eucalyptus, cultivated on marginal and degraded lands not usable for food production are attractive for their high productivity and resistance to biotic and abiotic stresses (Mariani et al., 2010; Fiorentino et al., 2013). Bioconversion of lignocellulosic material into valuable products requires a multi-step process including pretreatment of lignocelluose to remove lignin making the polysaccharides more accessible to the following steps of hydrolysis of the carbohydrates and fermentation of the resulting monosaccharides. Among saccharification methods, the use of microbial hydrolytic enzymes is the most eco-friendly system (Taherzadeh and Kamiri, 2007). However, the hydrolysis step represents one of the main bottlenecks in the process (Berrin et al., 2012), since enzymatic saccharification still remains the largest contributor to the overall cost of lignocellulose conversion, although a wide range of process configurations for conversion of lignocellulosic feedstock into fermentable sugars have been reported (Liguori et al., 2016). Therefore, the search for new microorganisms producing (hemi)cellulolytic enzymes more efficient in lignocellulose conversion represents one of the main routes to contribute to the cost reduction of enzymatic saccharification. Several studies on isolation of new (hemi)cellulolytic microorganisms have been reported, including bacteria belonging to *Clostridium* spp., *Cellulomonas* spp., *Bacillus* spp., *Thermomonospora* spp., *Ruminococcus* spp., *Erwinia* spp., *Acetovibrio* spp., *Microbispora* spp., and *Streptomyces* spp. (Sun and Cheng, 2002; Ventorino et al., 2015) and fungi such as *Sclerotium rolfsii, Phanerochaete chrysosporium, Trichoderma* spp., *Aspergillus* spp., *Schizophyllum* spp.,*Talaromyces* spp., and *Penicilium* spp. (Dos Reis et al., 2003; Jorgensen et al., 2003; Balat, 2011). Among these, filamentous bacteria belonging to class Actinobacteria are known to be able to produce enzymes for efficient decomposition of the polysaccharides of lignocellulose (Kirby, 2005; Berlemont and Martiny, 2013; Vetrovský et al., 2014). However, reducing the high cost of cellulase and xylanase enzymes for biomass hydrolysis still remains one of the main challenges for achieving economic feasibility of lignocellulose conversion.

In this context, we have previously investigated the dynamics of the microbiota during the natural biodegradation of lignocellulosic biomass crops (*Arundo donax, Eucalyptus camaldulensis*, and *Populus nigra*). These perennial biomass crops were characterized by a highly complex bacterial community, in which the most frequently occurring bacteria belonged to the phyla Actinobacteria, Proteobacteria, Bacteroidetes, and Firmicutes. Moreover, the three biomass crops were used to isolate new bacterial strains well-adapted to growth on cellulose as main carbon source (Ventorino et al., 2015). Among these isolates, 24 bacterial strains belonging to Actinobacteria were used in this work in order to select microorganisms producing biocatalysts able to hydrolyze the pretreated lignocellulose biomass of *A. donax*. The Actinobacteria strains were screened for their ability to secrete multiple enzyme activities as well as for their endo-cellulase production. Moreover, the strains producing the highest titers of endo-cellulase activities were subjected to partial enzyme characterization and tested in saccharification experiments using *A. donax* pretreated biomass. A proteomic approach was also used to identify the proteins putatively responsible for the best endo-cellulase producing strain.

## Materials and methods

### Bacterial strains, molecular identification and phylogenetic analysis

Twenty-four bacterial strains, belonging to the microbial collection of the Department of Agricultural Sciences, division of Microbiology of the University of Naples Federico II, were used in this study. These strains, previously isolated from lignocellulosic biomass of *A. donax, E. camaldulensis*, and *P. nigra* during biodegradation under natural conditions (Ventorino et al., 2015), were identified as Actinobacteria on the basis of their colony morphology, microscopic features (phase-contrast microscopy, shape, dimension, and presence of spores) and biochemical characteristics (Gram-stains, catalase activity).

Molecular identification was performed by 16S rRNA gene sequencing. Fast DNA SPIN kit for soil (MP Biomedicals, Illkirch Cedex, France) was used to extract and purify the total genomic DNA according to the supplier's recommendations. Approximately 50 ng of DNA was used as template for PCR assays. Synthetic oligonucleotide primers fD1 (5′-AGAGTTTGATCCTGGCTCAG-3′) and rD1 (5′-AAGGAGGTGATCCAGCC-3′) were used to amplify the 16S rRNA gene as reported by Palomba et al. (2011). The PCR conditions were as described by Ventorino et al. (2016a). Amplicons were purified using the QIA quick gel extraction kit (Qiagen S.p.A., Milan, Italy) after visualization by agarose (1.5% wt/vol) gel electrophoresis at 100 V for about 1 h. The DNA sequences were determined and analyzed as previously reported (Pepe et al., 2013a), and were compared to the 16S ribosomal RNA sequences database of GenBank nucleotide data library using the BLAST software at the National Centre of Biotechnology Information website (http://www.ncbi.nlm.nih.gov/Blast.cgi). Multiple nucleotide alignments of the nearly full-length 16S rRNA sequences of bacterial isolates and type strains within each of the defined species were carried out using the ClustalW program from MEGA version 4.0 (Tamura et al., 2007). The nucleotide sequences of the type strains were recovered from the Ribosomal Database Project (RDP—https://rdp.cme.msu.edu). The phylogenetic tree was inferred by the Neighbor-Joining method with the Maximum Composite Likelihood model in MEGA4 program with bootstrap values based on 1000 replications.

### Screening for enzymatic activities on solid media

Endo and exo-cellulase, cellobiase, xylanase, pectinase, ligninase, peroxidase, and laccase activities were evaluated in all strains by qualitative and semi-quantitative agar spot methods as previously described (Ventorino et al., 2015). Briefly, bacterial cell suspensions (0.5 of McFarland Turbidity Standard corresponding to approximately 1.5 × 10^8^ CFU mL^−1^) were spotted in triplicate on selective solid media. Cellulase, pectinase and laccase activities were recorded as the “Indices of Relative Enzyme Activity, (I_CMC_ or I_PEC_ or I_LAC)_ = diameter of clearing or halo zone/colony diameter” using carboxymethylcellulose (CMC) agar, Pectin agar or liquid basal medium (LBM) agar (supplemented with 1 g L^−1^ of 2.2′-azino-bis (3-ethylbenzothiazoline-6-sulphonic acid [ABTS]) media, respectively (Pepe et al., 2013b; Ventorino et al., 2015). Exo-cellulase activity was estimated by inoculating the strains on Avicel agar and observing the development of bacterial colonies after incubation. Cellobiase and xylanase activities were detected by observing a clear zone around the colonies after incubation on cellobiose agar (Ventorino et al., 2015) or Luria-Bertani (LB) agar medium (Oxoid, Milan, Italy) supplemented with 0.05% Remazol brilliant blue-R and a 0.5% solution of sonicated xylan (Sigma-Aldrich, Milan, Italy) as described by Ko et al. (2012). Ligninase activity was assayed using the culture medium guaiacol agar (CGA) supplemented with 2 g L^−1^ of alkali lignin (Okino et al., 2000) or 2 g L^−1^ of vegetable biomass powder from *A. donax* (Ventorino et al., 2015). Finally, LBM agar medium supplemented with 0.1 g L^−1^ of Azure-B was used to detect peroxidase activity as previously reported (Ventorino et al., 2015).

### Screening for endo-cellulase activity in liquid medium

The *Streptomyces* strains were grown on plate count agar (PCA) medium (0.5% tryptone, 0.25% yeast extract, 0.1% glucose, and 1.5% agar) for 14 days at 28°C. The pre-inoculum was prepared by adding one agar plug (4 mm) from a 2-week old plate into 10 mL of liquid medium containing 10 g L^−1^ carboxymethylcellulose (CMC), 0.5 g L^−1^ yeast extract, 2.5 g L^−1^ (NH_4_)_2_SO_4_, 2.7 g L^−1^ KH_2_PO_4_, 5.3 g L^−1^ Na_2_HPO_4_, 0.2 g L^−1^ NaCl, 0.2 g L^−1^ MgSO_4_, and 0.05 g L^−1^ CaCl_2_. After 48 h of incubation on a rotary shaker (250 rpm) at 28°C, the spore concentration was determined by using the Bürker-chamber through decimal serial dilutions in isotonic Ringer's solution. Fermentation was carried out in 100 mL plugged Erlenmeyer flasks, each containing 20 mL of the same medium used for the preinoculum. A quantity of 2 × 10^6^ spores mL^−1^ was inoculated. The flasks were incubated for 10 days at 28°C on a rotary shaker (200 rpm). Samples were withdrawn every 24 h for 10 days and the supernatants after removal of cells by three centrifugations (4629 × g, 30 min each at 4°C) used for measurement of extracellular endo-cellulase activity. The assay was performed by using Azo-CMC (Megazyme, Ireland) as substrate, following supplier's instructions and determined by referring to *Trichoderma* sp. endo-cellulase standard curve.

### Partial characterization of enzymes

The optimum temperature and pH of the cellulase activity of four selected *Streptomyces* strains was determined using the supernatant of bacterial cultures recovered and concentrated by ultrafiltration with a 10 kDa polyethersulfone membrane (Millipore Corporation, Bedford, MA, USA).

To assess the optimum temperature, the enzymatic activity assay was performed at 40, 45, 50, and 55°C by using Azo-CM-Cellulose (Azo-CMC; Megazyme, Ireland) dissolved in 50 mM Na citrate at pH 5.0 as substrate, following supplier's instructions.

To determine the optimum pH of cellulase activity, the experiments were performed at 40°C in 50 mM Na-citrate buffer, at pH 5.0 and 6.0, using Azo-CMC (Megazyme, Ireland) as substrate dissolved in the above-mentioned buffer, following supplier's instructions.

The reported results correspond to mean values of the three independent experiments each one performed in three replicates.

### Enzymatic hydrolysis of *Arundo donax* and determination of sugar content

*A. donax* biomass, pretreated according to Garbero et al. (2010) and De Bari et al. (2013) was utilized as substrate in biotransformation experiments carried out in capped tubes, on the rotary shaker ThermoMixer C (Eppendorf, Milan, Italy) at 50°C up to 72 h. The reaction mixture contained 50 mM sodium citrate buffer pH 5.0 or 6.0, the enzymatic cocktail and a concentration of pretreated biomass of 5% (w/v) in a total volume of 2.5 mL. The components of the enzymatic mix, Accelerase 1500, Accelerase BG and Accelerase XY, (Mix 1), were obtained from Genencor and were applied at the following loadings, expressed as units per gram of pretreated substrate: 5.4, 145, and 4000, respectively.

Enzymatic extracts endowed with endo-cellulase activity from four selected *Streptomyces argenteolus* strains were obtained by removal of cells through three subsequent centrifugations (5500 × g, 30 min each at 4°C) and concentration by using the stirred ultrafiltration Amicon® system (Millipore Corporation, Bedford, MA, USA) with a 10 kDa polyethersulfone membrane. These crude extracts were utilized in replacement of the commercial counterpart represented by the Accelerase 1500. Samples were collected at different time intervals (0, 48, and 72 h), cooled on ice, centrifuged at 16,500 × g for 30 min at 4°C and the amount of sugars released were quantified in the recovered supernatants, as described below. The saccharification yields, that are means of three replicates, were expressed as percentages with respect to the sugar content of the pretreated material before the hydrolysis (Garbero et al., 2010; De Bari et al., 2013).

The sugars released upon enzymatic hydrolysis were determined by high-performance liquid chromatography (HPLC) using a system 7Q (Dionex, California, USA), equipped with an anionic exchange column (Carbopac PA-100) and a pulsed electrochemical detector. Glucose and xylose were separated with 16 mM sodium hydroxide at a flow rate of 0.25 mL min^−1^, and identified by the respective standards. Fucose was used as internal standard.

### Determination of protein concentration

Protein concentration of crude enzyme preparation was determined by using Bradford reagent of Biorad (München, Germany) following supplier's instructions. Bovin serum albumin (BSA) was used to set up the standard curve.

### Enzyme identification

#### Crude extract preparation

Proteins secreted by the selected *S. argenteolus* strains were precipitated from the cultures corresponding to the maximum endo-cellulase production by the addition of ammonium sulfate up to 80% saturation, after removing cells by three centrifugations (4629 × g**, 30 min each at 4°C). Precipitated proteins were recovered by centrifugation at 7232 × g for 45 min at 4°C and transferred into in 50 mM Na_2_HPO_4_ pH 6.0, by diafiltration through Vivaspin® 20 in polyethersulfone (PES) with a cut-off of 10 kDa (Sartorius, Göttingen, Germany).

### Protein identification by mass spectrometry

Precipitated proteins were dissolved in 1 mL of Tris 300 mM pH 8.0, urea 6 M, EDTA 10 mM, and disulfide bridges were reduced with 1,4-Dithiothreitol (DTT) 10 mM (final concentration) at 37°C for 2 h and then cysteines alkylated by adding iodoacetamide (IAM) 100 mM (final concentration) at room temperature for 30 min in the dark. The protein sample was desalted by size exclusion chromatography on a Sephadex G25M column (GE Healthcare). Protein containing fractions were concentrated by evaporation under vacuum to 100 μL. Enzymatic digestion was then performed by adding 2 μg trypsin in a sample volume of 100 μL. After incubation at 37°C for 16 h, the surnatant was recovered by centrifugation at 45 × g, and the peptide mixture was filtered through a 0.22 μm PVDF membrane (Millipore), concentrated and purified using reverse phase C18 Zip Tip pipette tips (Millipore). Peptides were eluted with 20 μL of a solution made of 50% acetonitrile/0.1% formic acid in MilliQ water and analyzed by LC-MS/MS. LC-MSMS analyses were carried out on a 6520 Accurate-Mass Q-TOF LC/MS System (Agilent Technologies, Palo Alto, CA) equipped with a 1200 HPLC system and a chip cube (Agilent Technologies). After loading 10% of the recovered sample, the peptide mixture was first concentrated and washed in 40 nL enrichment column (Agilent Technologies chip), with 0.1% formic acid in 2% acetonitrile as the eluent. Peptides were then fractionated on a C18 reverse-phase capillary column (Agilent Technologies chip) at a flow rate of 400 nL min^−1^, with a linear gradient of eluent B (0.1% formic acid in 95% acetonitrile) in A (0.1% formic acid in 2% acetonitrile) from 7 to 80% in 50 min. Data analysis was performed using data-dependent acquisition of one MSscan (mass range from 300 to 1800 m/z) followed by MS/MS scans of the five most abundant ions in each MS scan. MS/MS spectra were measured automatically when the MS signal surpassed the threshold of 50,000 counts. Double and triple charged ions were preferably isolated and fragmented. The acquired MS/MS spectra were transformed in Mascot generic format (.mgf) and used for protein identification in the unreviewed set of protein entries that are present in the NCBInr database for Actinobacteria, with a licensed version of MASCOT software (www.matrixscience.com) version 2.4.0.

Additional MASCOT search parameters were: peptide mass tolerance 10 ppm, fragment mass tolerance 0.6 Da, allowed missed cleavages up to 3, carbamidomethylation of cysteines as fixed modification, oxidation of methionine, and pyro-Glu N-term Q, as variable modifications. Only doubly and triply charged ions were considered. Ions score was −10 log (P), where P is the probability that the observed match is a random event. Individual ion scores >43 indicated identity or extensive homology (*P* < 0.05). Protein scores were derived from ion scores as a non-probabilistic basis for ranking protein hits (http://www.matrixscience.com/help/interpretation_help.html).

Trypsin, dithiothreitol, iodoacetamide and NH_4_HCO_3_ were purchased from Sigma-Aldrich. All other reagents and solvents were of the highest purity available from Baker (Mumbai, MH, India).

### Accession numbers

The 16S rRNA gene sequences obtained from bacterial strains were deposited in the GenBank nucleotide database under accession numbers from KX431234 to KX431257 (http://www.ncbi.nlm.nih.gov).

### Statistical analyses

One-way ANOVA followed by Tukey's HSD *post-hoc* for pair-wise comparison of means (at *P* < 0.05) was used to assess the difference in the enzymatic activities of bacterial strains such as I_CMC_, I_PEC_, and Azo-CMCase as well as in the glucose and xylose yields (%). Statistical analyses were performed using SPSS 19.0 statistical software package (SPSS Inc., Cary, NC, USA).

## Results

### Phenotypic characterization and molecular identification of microbial strains

**Figure 1:**
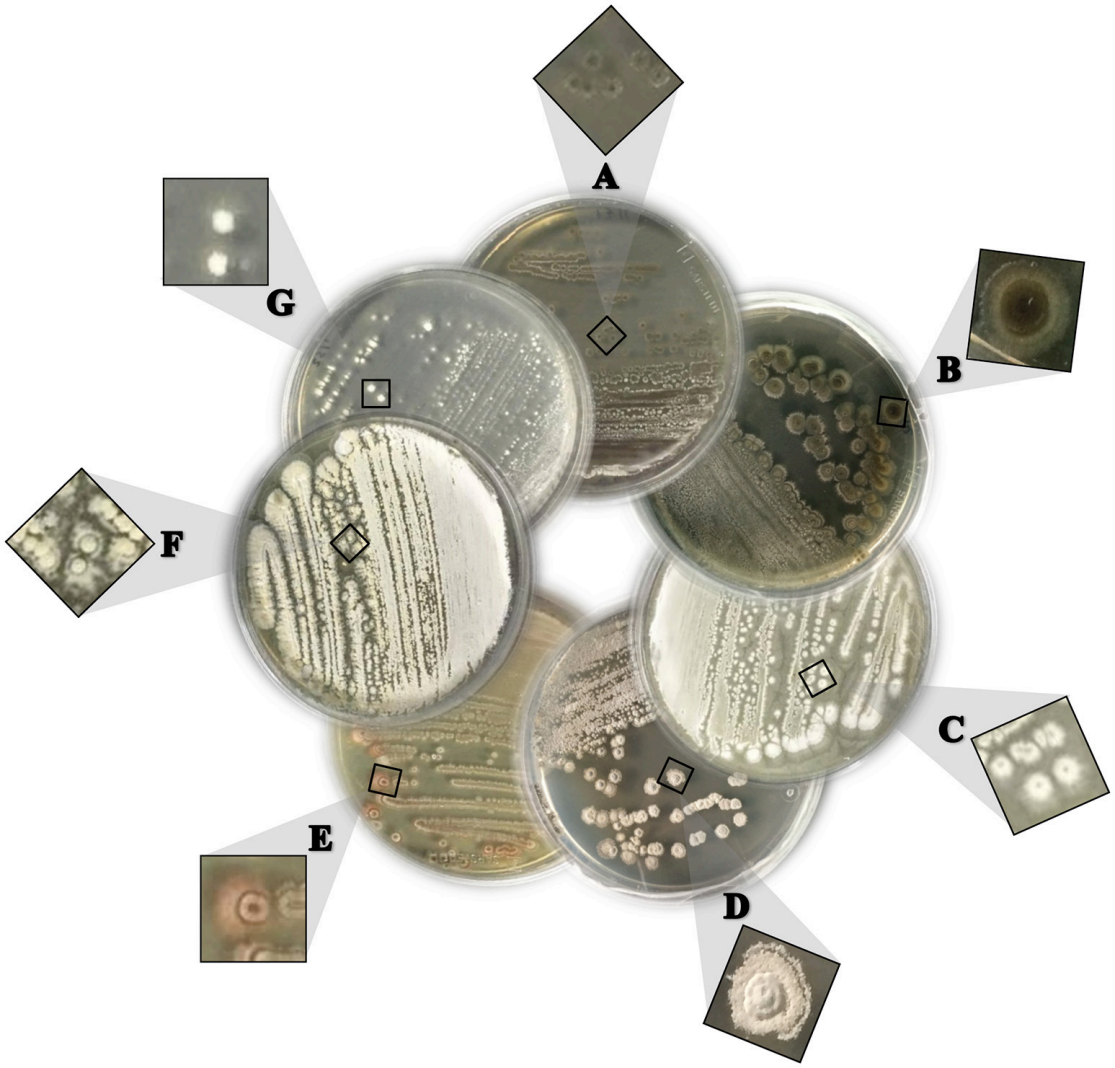
**Seven different phenotypes (A–G)** based on the colony morphology of the representative *Streptomyces* spp. strains grown on starch casein agar medium.

Twenty-four lignocellulosic-adapted bacterial strains, with potential enzymatic degradation activities on lignocellulosic biomass, were grown on starch casein agar medium and characterized from a phenotypic point of view by analysis of colony and cell morphology, presence of spores, gram reaction, and catalase activity. All strains were Gram and catalase positive and produced a spore-bearing aerial mycelium. They were identified as *Actinobacteria* and grouped in seven phenotypes on the basis of colony morphology (Figure 1).

As shown in Table 1, the strains were identified by 16SrRNA gene sequencing showing that all strains belonged to the genus *Streptomyces* (*S*.). In particular, the most highly represented species was *S. argenteolus* representing 50% of strains (phenotype A), while eight strains were identified as members of species *Streptomyces flavogriseus* (synonym *S. flavovirens*) (phenotype B) and *Streptomyces fimicarius* (synonyms *S. acrimycini, S. baarnensis, S. caviscabies*, and *S. flavofuscus*) (phenotype C). Finally, the other 4 strains belonged to the species *Streptomyces drozdowiczii, Streptomyces rubrogriseus, Streptomyces albolongus*, and *Streptomyces ambofaciens* (phenotypes D, E, F, and G).

**Table 1:** **Phenotypic characterization and molecular identification of 24 Actinobacteria strains isolated from lignocellulosic biomasses of *Arundo donax, Eucalyptus camaldulensis* and *Populus nigra***.

**Strain**	**Source**	**Colony morphology**	**Cell morphology**	**Gram reaction**	**Catalase**	**Phenotype**	**Identification (% identity)**	**Accession number**
AP71C	*P. nigra*	Irregular, silver, flat, erose, opaque	Spore-bearing hyphae	+	+	A	*Streptomyces argenteolus* (99%)	KX431256
AE612P	*E. camaldulensis*	Irregular, silver, flat, erose, opaque	Spore-bearing hyphae	+	+	A	*Streptomyces argenteolus* (100%)	KX431240
AE58P	*E. camaldulensis*	Irregular, silver, flat, erose, opaque	Spore-bearing hyphae	+	+	A	*Streptomyces argenteolus* (100%)	KX431241
AA63A	*A. donax*	Irregular, silver, flat, erose, opaque	Spore-bearing hyphae	+	+	A	*Streptomyces argenteolus* (100%)	KX431246
AE61P	*E. camaldulensis*	Irregular, silver, flat, erose, opaque	Spore-bearing hyphae	+	+	A	*Streptomyces argenteolus* (100%)	KX431247
AA64C	*A. donax*	Irregular, silver, flat, erose, opaque	Spore-bearing hyphae	+	+	A	*Streptomyces argenteolus* (100%)	KX431248
AA85A	*A. donax*	Irregular, silver, flat, erose, opaque	Spore-bearing hyphae	+	+	A	*Streptomyces argenteolus* (100%)	KX431249
AP73A	*P. nigra*	Irregular, silver, flat, erose, opaque	Spore-bearing hyphae	+	+	A	*Streptomyces argenteolus* (99%)	KX431251
AP51A	*P. nigra*	Irregular, silver, flat, erose, opaque	Spore-bearing hyphae	+	+	A	*Streptomyces argenteolus* (100%)	KX431243
AE710A	*E. camaldulensis*	Irregular, silver, flat, erose, opaque	Spore-bearing hyphae	+	+	A	*Streptomyces argenteolus* (100%)	KX431244
AE82P	*E. camaldulensis*	Irregular, silver, flat, erose, opaque	Spore-bearing hyphae	+	+	A	*Streptomyces argenteolus* (100%)	KX431242
AP64C	*P. nigra*	Irregular, silver, flat, erose, opaque	Spore-bearing hyphae	+	+	A	*Streptomyces argenteolus* (100%)	KX431237
AA62C	*A. donax*	Irregular, black/gray, slightly raised, lobate, opaque	Spore-bearing hyphae	+	+	B	*Streptomyces flavogriseus* (99%)	KX431257
AE53P	*E. camaldulensis*	Irregular, black/gray, slightly raised, lobate, opaque	Spore-bearing hyphae	+	+	B	*Streptomyces flavogriseus* (100%)	KX431238
AA714A	*A. donax*	Irregular, black/gray, slightly raised, lobate, opaque	Spore-bearing hyphae	+	+	B	*Streptomyces flavogriseus* (99%)	KX431253
AA72A	*A. donax*	Irregular, black/gray, slightly raised, lobate, opaque	Spore-bearing hyphae	+	+	B	*Streptomyces flavogriseus* (99%)	KX431252
AA88P	*A. donax*	Irregular, white, flat, erose, opaque	Spore-bearing hyphae	+	+	C	*Streptomyces fimicarius* (100%)	KX431236
AE78P	*E. camaldulensis*	Irregular, white, flat, erose, opaque	Spore-bearing hyphae	+	+	C	*Streptomyces fimicarius* (100%)	KX431245
AE73P	*E. camaldulensis*	Irregular, white, flat, erose, opaque	Spore-bearing hyphae	+	+	C	*Streptomyces fimicarius* (100%)	KX431239
AE618X	*E. camaldulensis*	Irregular, white, flat, erose, opaque	Spore-bearing hyphae	+	+	C	*Streptomyces fimicarius* (99%)	KX431255
AA86P	*A. donax*	Irregular, white/gray, raised, undulate, opaque	Spore-bearing hyphae	+	+	D	*Streptomyces drozdowiczii* (100%)	KX431234
AA74P	*A. donax*	Irregular, red/white, flat, erose, opaque	Spore-bearing hyphae	+	+	E	*Streptomyces rubrogriseus* (100%)	KX431235
AP71X	*P. nigra*	Irregular, white/beige, raised, lobate, opaque	Spore-bearing hyphae	+	+	F	*Streptomyces albolongus* (99%)	KX431250
AE66P	*E. camaldulensis*	Irregular, white, flat, erose, opaque	Spore-bearing hyphae	+	+	G	*Streptomyces ambofaciens* (99%)	KX431254

**Figure 2:**
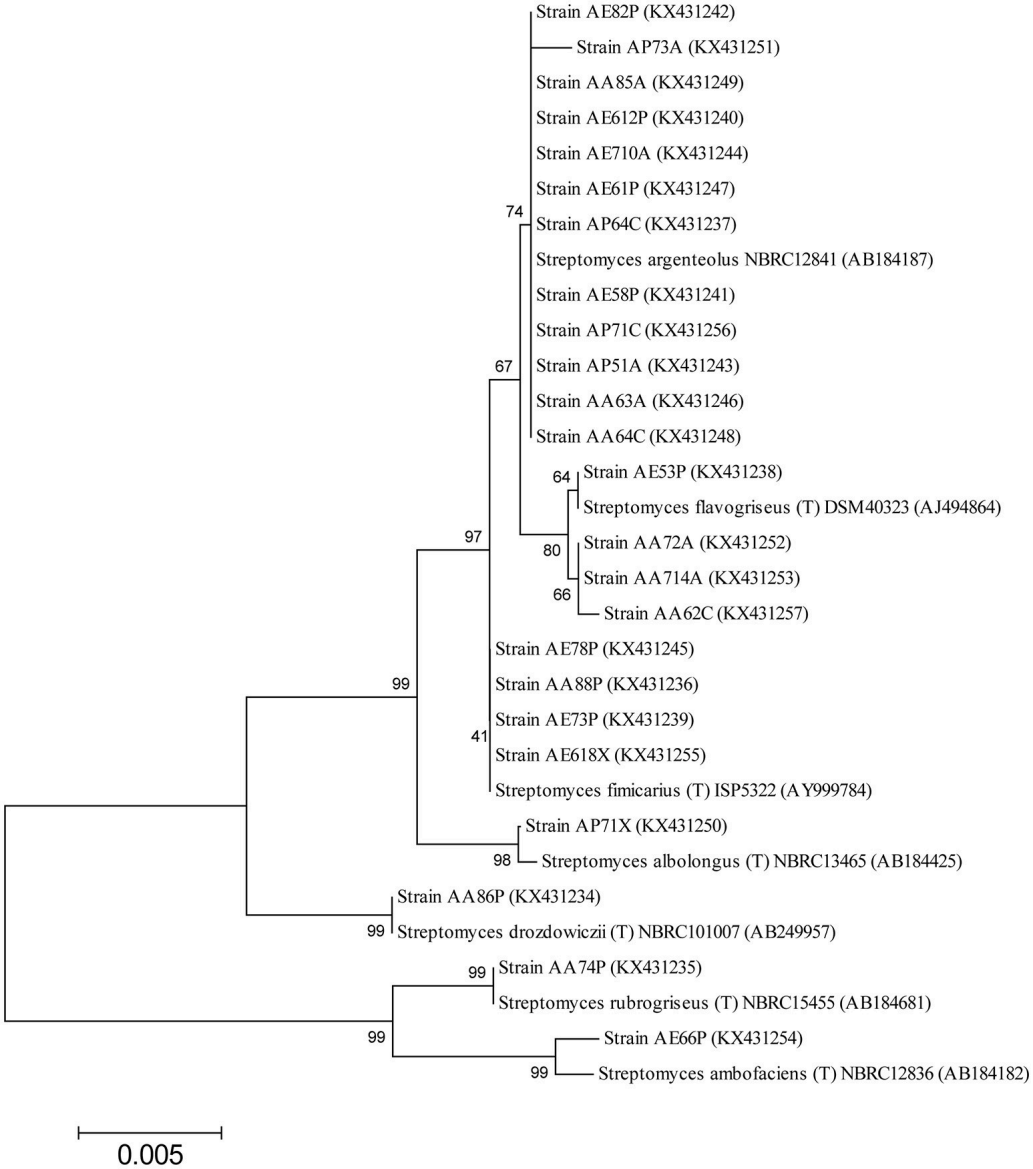
**Neighbor-Joining tree based on the comparison of 16S rRNA gene sequences showing the relationships among *Streptomyces* strains**. Bootstrap values (expressed as percentages of 1000 replications) are given at the nodes. The sequence accession numbers used for the phylogenetic analysis are shown in parentheses following the species name. Strains marked with “(T)” represent type strains. The *scale bar* estimates the number of substitutions per site.

The nearly full-length gene sequences (>1400 bp) of the *Streptomyces* bacteria identified were grouped by phylogenetic analysis into seven different clusters, generating a *consensus* tree. The phylogenetic tree was generated from the distance data using the Neighbor-Joining method with the Maximum Composite Likelihood model in the MEGA4 Program (Figure 2). The nucleotide sequences of related type strains of different species were included in the data set. High bootstrap values were observed and indicated significant branching points in the phylogenetic tree. Strains representative of the dominant species of *S. argenteolus* were placed in a cluster with bootstrap values higher than 70%. The other species were included in clusters with bootstrap values from 80 to 99%, except for the cluster of strains identified as *S. fimicarius* (41%, Figure 2).

### Screening by biotechnological characterization

The *Streptomyces* strains were assayed for different enzymatic activities specific for the degradation of the lignocellulosic biomass using qualitative and semi-quantitative screening. One hundred percent of the bacterial strains exhibited multiple enzymatic activities (from three to six) as shown in Table 2. Endo-cellulase activity was highlighted in all *Streptomyces* strains with I_CMC_ values ranging from 20 to 40. In particular, five strains (*S. argenteolus* AE58P, *S. albolongus* AP71X, *S. argenteolus* AE82P, *S. argenteolus* AA85A, and *S. argenteolus* AP51A) showed the highest activity with I_CMC_ > 30 (*P* < 0.05). Furthermore, 16 strains showed endo-cellulase activity between 25 ≤ I_CMC_ ≤ 30, whereas three other strains (*S. argenteolus* AP73A, *S. flavogriseus* AA72A, and *S. argenteolus* AP64C) showed I_CMC_ <25. Moreover, all bacterial strains showed also exo-cellulase activity, since were able to grow on culture medium containing microcrystalline cellulose (Avicel) as sole carbon source, and 22 *Streptomyces* strains exhibited pectinase activity with I_PEC_ ≥ 10 (Table 2). The highest pectinase activity was observed in the strains *S. argenteolus* AE82P and *S. argenteolus* AA63A (I_PEC_ = 28, *P* < 0.05).

**Table 2:** **Multi-enzymatic activity of 24*Streptomyces* spp. strains isolates from lignocellulosic biomasses of *Arundo donax, Eucalyptus camaldulensis* and *Populus nigra***.

**Strain**	C^*^	**A^§^**	**CE^†^**	P^*^	**X^†^**	AB^*^	**AZ^†^**	**L^†^**	**AD^†^**
*Streptomyces argenteolus* AE58P	40 ± 1.0^a^	+	−	22 ± 1.0^cd^	+	−	−	−	−
*Streptomyces albolongus* AP71X	36 ± 0.5^b^	++	−	−	++	−	−	−	−
*Streptomyces argenteolus* AE82P	32 ± 0.0^c^	+	−	28 ± 0.6^a^	++	−	−	−	−
*Streptomyces argenteolus* AA85A	32 ± 1.0^c^	+	−	26 ± 0.6^b^	+	−	−	−	−
*Streptomyces argenteolus* AP51A	32 ± 0.5^c^	+	−	26 ± 0.0^b^	++	20 ± 0.3^a^	−	−	−
*Streptomyces rubrogriseus* AA74P	30 ± 0.0^d^	+	−	20 ± 1.0^ef^	+	−	−	−	−
*Streptomyces fimicarius* AA88P	30 ± 0.6^de^	+	+/−	18 ± 0.6^f^	+/−	−	−	−	−
*Streptomyces flavogriseus* AE53P	30 ± 0.5^d^	+	−	10 ± 0.3^l^	−	−	−	−	−
*Streptomyces fimicarius* AE73P	30 ± 0.0^d^	+	+	14 ± 1.0^i^	++	−	−	−	−
*Streptomyces argenteolus* AE612P	30 ± 0.6^de^	+	−	22 ± 0.3^cd^	++	−	−	−	−
*Streptomyces argenteolus* AA63A	30 ± 0.6^de^	+	+	28 ± 0.0^a^	+	−	−	−	−
*Streptomyces argenteolus* AE61P	30 ± 0.5^d^	+	−	20 ± 0.0^ef^	−	−	−	−	−
*Streptomyces argenteolus* AA64C	30 ± 0.6^de^	+	−	22 ± 1.0^cd^	+	−	−	−	−
*Streptomyces fimicarius* AE618X	30 ± 0.0^d^	+	+	22 ± 0.5^cd^	++	−	−	−	−
*Streptomyces ambofaciens* AE66P	30 ± 0.5^d^	+	+/−	-	+/−	−	−	−	−
*Streptomyces drozdowiczii* AA86P	28 ± 0.6^fg^	+	++	22 ± 0.6^c^	+	−	−	−	−
*Streptomyces flavogriseus* AA62C	28 ± 0.6^fg^	+	+	16 ± 0.6^gh^	+	−	+	−	−
*Streptomyces fimicarius* AE78P	28 ± 0.0^ef^	+	+	20 ± 1.0^ef^	++	−	−	−	−
*Streptomyces argenteolus* AP71C	26 ± 0.5^gh^	+	−	20 ± 0.6^de^	+	−	−	−	−
*Streptomyces argenteolus* AE710A	26 ± 0.0^gh^	++	+/−	18 ± 0.3^fg^	+	−	−	−	−
*Streptomyces flavogriseus* AA714A	26 ± 1.0^gh^	++	+	10 ± 0.0^l^	+	−	−	−	−
*Streptomyces argenteolus* AP73A	24 ± 0.6^hi^	++	+	22 ± 0.6^c^	+	−	−	−	−
*Streptomyces flavogriseus* AA72A	24 ± 0.5^i^	++	−	16 ± 0.6^gh^	+	20 ± 0.5^a^	−	−	−
*Streptomyces argenteolus* AP64C	20 ± 0.0^l^	+	+/−	16 ± 0.0^h^	+	−	−	−	−

Enzymatic activities: C, endo-cellulase; A, exo-cellulase; CE, cellobiase; X, xylanase; P, pectinase; AB, laccase; AZ, peroxidase; L, ligninase with guaiacol and lignin alkali; AD, ligninase with guaiacol and Arundo donax;

* I_CMC_ oI_PEC_ oI_LAC_ index, values represent the means ± SD of three replicates. Different letters after values indicate significant differences (P < 0.05);

§ growth;

† *−negative; + low intensity; ++ middle intensity*.

Xylanase activity was detected in approximately 80% of bacterial strains, whereas only eight strains were able to hydrolyze cellobiose.

Finally, the two strains identified as *S. argenteolus* AP51A and *S. flavogriseus* AA72A showed a high laccase activity (I_LAC_ = 20) in LBM agar medium supplemented with ABTS, whereas peroxidase activity was detected only in the strain *S. flavogriseus* AA62C. Lignin hydrolysis was not detected in any of the strains tested (Table 2).

### Screening of endo-cellulolytic microorganisms in liquid medium

A further screening of the 24 lignocellulosic-adapted *Streptomyces* strains was carried out in liquid medium containing the sole carbon source CMC as inducer substrate for endo-1,4-ß-glucanases. In Table 3, the values of the maximum AZO-CMCase activity measured for each strain and the corresponding time of production are reported. This screening confirmed three of the five *Streptomyces* strains with the highest activity in solid medium (*S. argenteolus* AE58P, *S. argenteolus* AE82P, and *S. argenteolus* AP51A) as the best endo-cellulase producers in liquid medium, whilst *S. albolongus* AP71X and *S. argenteolus* AA85A strains proved to be less effective producers than on solid medium. The *S. argenteolus* AE58P produced the highest endo-1,4-ß-glucanase activity level (0.41 ± 0.05 U mL^−1^; *P* < 0.05) after 6 days of fermentation and was chosen as reference. The maximum values of endo-cellulase production for *S. argenteolus* AP51A, *S. argenteolus* AE710A, *S. argenteolus* AE82P, and *S. argenteolus* AE612P were at least 50% in comparison to the maximum value shown by *S. argenteolus* AE58P. These strains were classified as high producers. The strains *S. argenteolus* AP73A, *S. fimicarius* AA88P, *S. flavogriseus* AA62C, *S. drozdowiczii* AA86P, *S. argenteolus* AP71C, *S. argenteolus* AE61P, *S. flavogriseus* AA72A, *S. fimicarius* AE78P showed a maximum value of activity between 34 and 45 % in comparison to the reference value. The other strains produced an endo-cellulase activity less than 34% of the reference.

**Table 3:** **Maximum value of Azo-CMCase activity measured for each strain and the corresponding time of production**.

**Bacterial Strain**	Maximum value of AZO-CMCase activity^*^ (U mL^−1^)	**Time (days)**
*Streptomyces argenteolus* AE58P	0.42 ± 0.04^a^	6
*Streptomyces argenteolus* AP51A	0.31 ± 0.07^b^	7
*Streptomyces argenteolus* AE710A	0.30 ± 0.05^b^	4
*Streptomyces argenteolus* AE82P	0.29 ± 0.07^b^	5
*Streptomyces argenteolus* AE612P	0.21 ± 0.04^bc^	5
*Streptomyces argenteolus* AP73A	0.19 ± 0.00^cd^	4
*Streptomyces fimicarius* AA88P	0.18 ± 0.03^cde^	3
*Streptomyces flavogriseus* AA62C	0.17 ± 0.04^cde^	7
*Streptomyces drozdowiczii* AA86P	0.16 ± 0.02^cde^	8
*Streptomyces argenteolus* AP71C	0.16 ± 0.03^cde^	4
*Streptomyces flavogriseus* AA72A	0.16 ± 0.04^cde^	6
*Streptomyces argenteolus* AE61P	0.16 ± 0.04^cde^	4
*Streptomyces fimicarius* AE78P	0.15 ± 0.05^cde^	7
*Streptomyces argenteolus* AA64C	0.14 ± 0.03^cde^	7
*Streptomyces argenteolus* AA85A	0.14 ± 0.02^cde^	4
*Streptomyces rubrogriseus* AA74P	0.13 ± 0.02^cde^	4
*Streptomyces argenteolus* AP64C	0.13 ± 0.03^cde^	6
*Streptomyces ambofaciens* AE66P	0.11 ± 0.00^de^	4
*Streptomyces albolongus* AP71X	0.11 ± 0.00^de^	4
*Streptomyces flavogriseus* AA714A	0.11 ± 0.02^de^	3
*Streptomyces flavogriseus* AE53P	0.10 ± 0.00^de^	4
*Streptomyces fimicarius* AE618X	0.10 ± 0.02^de^	3
*Streptomyces argenteolus* AA63A	0.10 ± 0.03^de^	4
*Streptomyces fimicarius* AE73P	0.08 ± 0.03^e^	4

* *The values represent the means ± SD of three replicates of three independent experiments. Different letters after the values indicate significant differences (P < 0.05)*.

### Partial characterization of enzymes

In order to evaluate the best conditions for long-term storage, the residual enzymatic activity of the best five endo-cellulase producing strains (*S. argenteolus* AE58P, *S. argenteolus* AP51A, *S. argenteolus* AE710A, *S. argenteolus* AE82P, and *S. argenteolus* AE612P) were assayed after up to 30 days of storage at 4, −20 and −80°C. The *S. argenteolus* AE710A retained most of the endo-1,4-ß-glucanase activity after 30 days (60% at 4°C and more than 80% at −20 and −80°C). Moreover, the *S. argenteolus* AE58P preserved 50–60% enzymatic activity after 30 days at all three tested temperatures, while the *S. argenteolus* AE82P and *S. argenteolus* AP51A strains retained 80–100% of activity at −20 and −80°C up to 30 days and lost 50% activity after 6 days at 4°C. By contrast, the tested storage conditions were not suitable for the endo-cellulase(s) produced by the *S. argenteolus* AE612P (data not shown). Based on these results, this strain was not subjected to further experiments.

**Figure 3:**
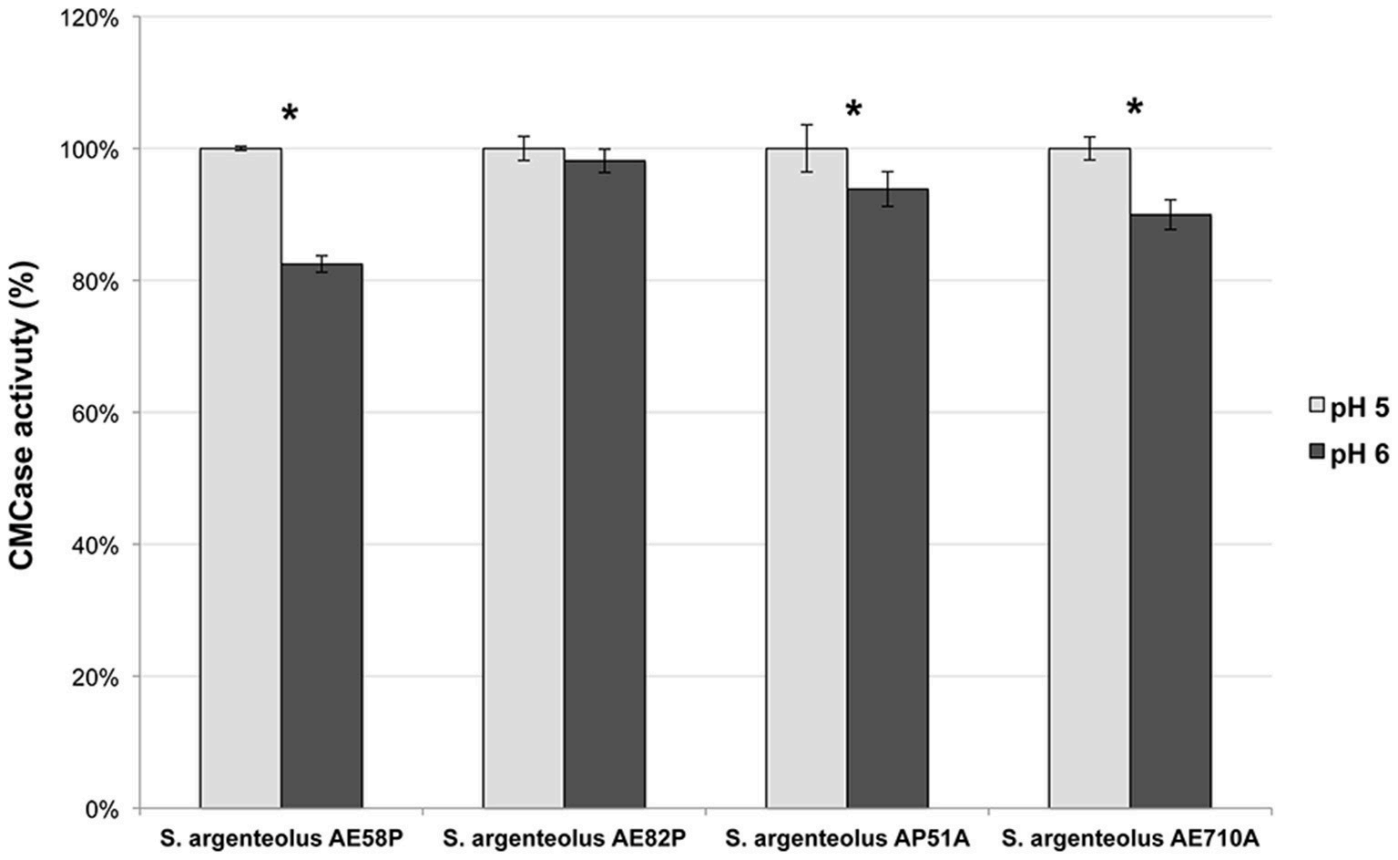
**Effect of two different pH values (5 and 6) on endo-cellulase activity produced by the selected strains *S. argenteolus* AE58P, AE82P, AP51A, and AE710A**. Bars indicate ± *SD* of three replicates of three independent experiments.^*^significant differences (*P* < 0.05; *t-*test) between the two conditions of the same strain.

**Figure 4:**
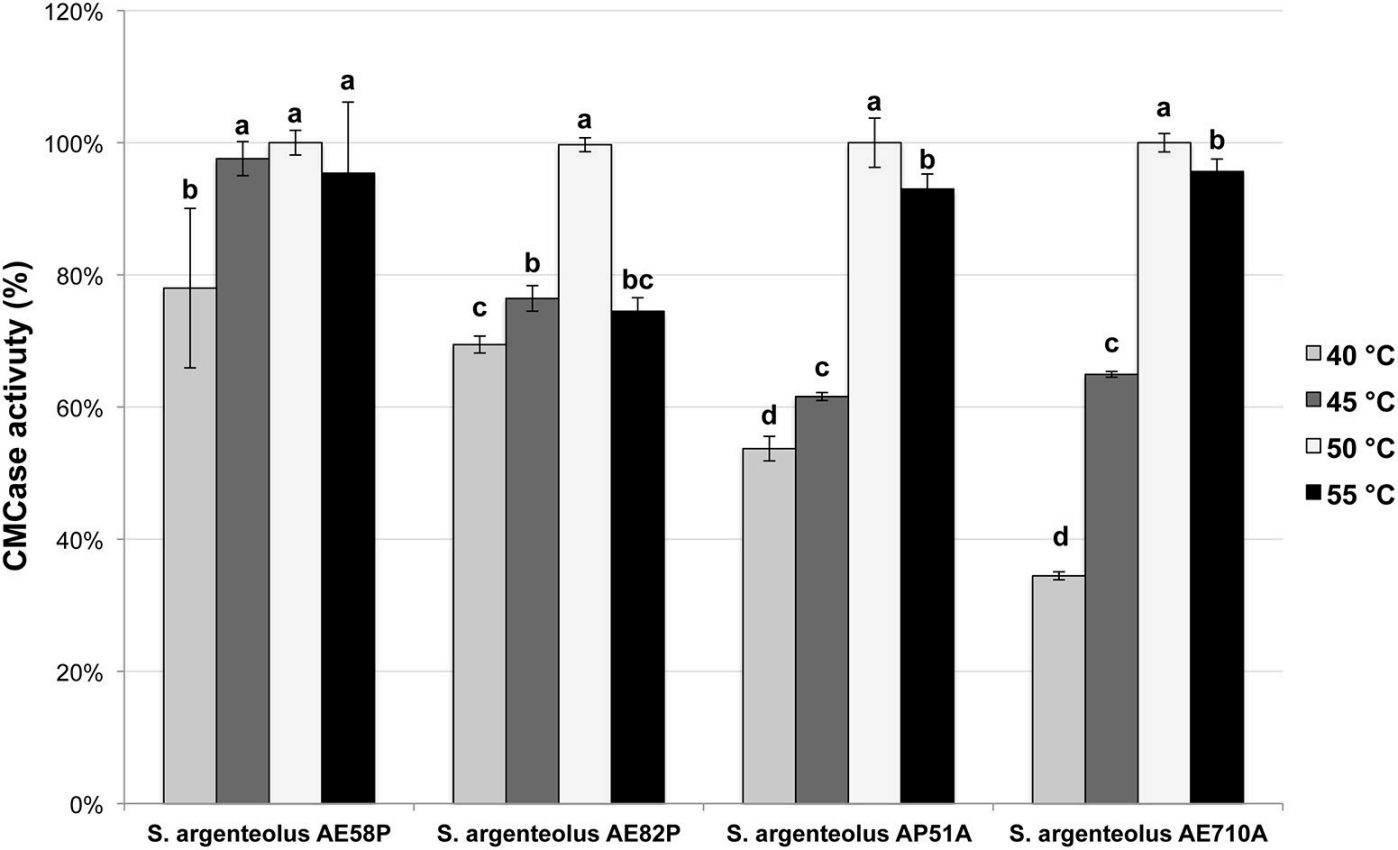
**Effect of different temperature on endo-cellulase activity produced by the selected strains of *S. argenteolus* AE58P, AE82P, AP51A, and AE710A**. Bars indicate ± *SD* of three replicates of three independent experiments. Different letters indicate significant differences (*P* < 0.05).

The optimal temperature and pH of endo-cellulase activities produced by *S. argenteolus* AE58P, *S. argenteolus* AP51A, *S. argenteolus* AE710A, and *S. argenteolus* AE82P were also evaluated. In the Na-citrate buffer, the cellulase activity produced by the analyzed *Streptomyces* strains showed an optimum at same pH value (5.0) (Figure 3). Furthermore, the optimum temperature for all the strains was 50°C (Figure 4), which represents the condition mostly used for the enzymatic hydrolysis of lignocellulosic biomasses.

### *Arundo donax* saccharification

Enzymatic crude extracts of the four *S. argenteolus* strains (AE58P, AE710A, AE82P, and AP51A) selected as the best endo-cellulase producers in liquid medium were concentrated by ultrafiltration and adopted to perform saccharification experiments on pretreated *A. donax* whose macromolecular composition in terms of percentage of glucans, xylans, and Klason lignin, was 36, 20, and 21%, respectively. The different degrees of cellulose and xylan hydrolysis were evaluated by determining the kinetics of glucose and xylose release during a 72 h incubation of the pretreated *A. donax* biomass in the presence of cellulose degrading enzymes (cellulase and β-glucosidase) and xylan related activities (xylanase and β-xylosidase). To this end the monosaccharide yields were evaluated on samples collected at different time intervals (0, 48, and 72 h). Cellulose and xylan conversion, when conducted for 72 h at 50°C in the presence of the commercial (hemi)cellulolytic activities from Genencor (Accellerase 1500) (see Mix 1 composition under Materials and Methods Section and Table 4), gave glucose and xylose yields of 30.17 and 68.9%, respectively. The replacement of the cellulolytic commercial preparation with the endo-cellulase from *S. argenteolus* AE58P (Mix 2) allowed glucose yield to reach, after 72 h, almost 76% of that obtained in the presence of the commercial counterpart after the same incubation time (Table 4). Significantly lower glucose yields (%) were obtained after 72 h when cellulases from *S. argenteolus* AE710A and *S. argenteolus* AE82P (Mix 3 and 5, Table 4) were utilized instead of the activities from the strain *S. argenteolus* AE58P. The most limited glucan hydrolysis was reached with the preparation from *S. argenteolus* AP51A (Mix 4, Table 4) and accounted for 43.6% of the maximum value (22.05%) reached with Mix 2. Moreover, two-fold increase in protein loading for the biocatalysts produced by all strains analyzed (Mix 2A, 3A, 4A, and 5A, Table 4) did not cause additional benefits to cellulose conversion. Also for the xylan hydrolysis the significant highest yields were obtained with the enzymatic extract from *S. argenteolus* AE58P (Mix 2) that accounted for 74% of the value obtained with the commercial biocatalysts (68.9% reached with Mix 1).

**Table 4:** **Glucose and xylose yields (%) during enzymatic hydrolysis of pretreated *Arundo donax* biomass by using commercial enzymes and cellulase from the four selected strains *S. argenteolus* AE58P, *S. argenteolus* AE82P, *S. argenteolus* AP51A, and *S. argenteolus* AE710A**.

Composition^*^	**Glucose yield (%)**		**Xylose yield (%)**	
	**48 h**	**72 h**	**48 h**	**72 h**
Mix 1	25.22 ± 1.50^b^	30.17 ± 0.81^a^	39.20 ± 8.92^cd^	68.90 ± 5.43^a^
Mix 2	15.83 ± 0.90^c^	22.05 ± 2.41^b^	38.10 ± 1.74^cde^	51.20 ± 3.50^bc^
Mix 2A	14.27 ± 1.72^cd^	22.98 ± 1.31^b^	35.01 ± 2.02^def^	53.10 ± 1.25^b^
Mix 3	10.22 ± 1.31^de^	11.00 ± 0.41^de^	22.81 ± 4.55^f^	27.12 ± 7.01^def^
Mix 3A	8.66 ± 2.51^e^	10.33 ± 1.46^de^	22.51 ± 2.33^f^	26.93 ± 6.22^def^
Mix 4	8.33 ± 0.33^e^	9.61 ± 0.05^e^	20.52 ± 6.10^f^	25.77 ± 3.25^ef^
Mix 4A	7.61 ± 0.44^e^	8.11 ± 0.41^e^	24.10 ± 2.72^f^	26.00 ± 1.93^def^
Mix 5	10.44 ± 0.96^de^	11.02 ± 2.21^de^	28.50 ± 4.09^def^	28.71 ± 3.90^def^
Mix 5A	10.27 ± 0.44^de^	10.83 ± 1.23^de^	28.31 ± 0.56^def^	29.90 ± 2.25^def^

* Composition of mix enzymes used:

Mix 1, Accellerase 1500 (5.4 U/g of pretreated biomass), Accellerase BG (145 U/g), Accellerase XY (4000 U/g);

Mix 2, cellulase from S. argenteolus AE58P (5.4 U/g), Accellerase BG (145 U/g), Accellerase XY (4000 U/g);

Mix 2A, cellulase from S. argenteolus AE58P (10.8 U/g), Accellerase BG (145 U/g), Accellerase XY (4000 U/g);

Mix 3, cellulase from S. argenteolus,AE82P (5.4 U/g), Accellerase BG (145 U/g), Accellerase XY (4000 U/g);

Mix 3A, cellulase from S. argenteolus AE82P (10.8 U/g), Accellerase BG (145 U/g), Accellerase XY (4000 U/g);

Mix 4, cellulase from S. argenteolus AP51A (5.4 U/g), Accellerase BG (145 U/g), Accellerase XY (4000 U/g);

Mix 4A, cellulase from S. argenteolus AP51A (10.8 U/g), Accellerase BG (145 U/g), Accellerase XY (4000 U/g);

Mix 5, cellulase from S. argenteolus AE710A (5.4 U/g), Accellerase BG (145 U/g), Accellerase XY (4000 U/g);

*Mix 5A, cellulase from S. argenteolus AE710A (10.8 U/g), Accellerase BG (145 U/g), Accellerase XY (4000 U/g). The values represent the means ± SD of three replicates*.

*Different letters after the values indicate significant differences (P < 0.05) in glucose yield or xylose yield*.

In order to improve the glucose and xylose yields, biotransformation experiments with Mix 1 and 2 were performed at pH 6.0 but the maximum glucose yields obtained after 72 h of incubation (29.50 and 13.88%) were lowered by 13.5 and 44 %, respectively compared to the result obtained at pH 5.0 (data not shown).

### Endo-cellulase(s) identification, sequence(s) of the cellulase gene(s) and its derived proteins

Due to the promising results obtained by using the enzymatic crude extracts from *S. argenteolus* AE58P in the pretreated *A. donax* saccharification experiments, the proteins putatively responsible for endo-cellulase activity in this strain were tentatively identified by proteomic strategy.

The secreted proteins from the above mentioned strain were precipitated by the addition of ammonium sulfate, recovered and diafiltered in 50 mM Na_2_HPO_4_, pH 6.

The peptide mixture obtained by enzymatic digestion by trypsin was analyzed by LC-MS/MS and query of the NCBInr database for *Actinobacteria* with a licensed version of MASCOT software (www.matrixscience.com). The identified proteins are reported in Table 5.

**Table 5:** **Mascot search results of LC-MS/MS data against NCBInr database showing identified proteins' score, number of peptides, and sequence coverage**.

**ID NCBInr (gi number)**	**Protein name**	**Score**	**Number of peptides**	**Sequence coverage (%)**
669633175	Cellulase, family GH12 [*Streptomyces argenteolus*]	267	5	20%
669633167	Cellulase, family GH5 [*Streptomyces argenteolus*]	229	4	9%
503810539	MULTISPECIES: glycosyl hydrolase [*Streptomyces*]	155	4	7%
503919052	MULTISPECIES: glycosyl hydrolase family 5 [*Streptomyces*]	135	3	8%
503918330	Glycosyl hydrolase family 5 [*Streptomyces flavogriseus*]	123	3	4%
493425639	Cellulose binding domain protein [*Streptomyces turgidiscabies*]	106	3	8%
443340544	Putative Secreted cellulose-binding protein [*Streptomyces viridochromogenes* Tue57]	69	2	8%
478750613	Cellulose-binding protein [*Streptomyces* sp. PAMC26508]	44	2	13%

Several proteins were confidently identified in different *Streptomyces* spp., eight of which belong to the class of Carbohydrate active enzymes. In detail, three proteins were annotated as containing carbohydrate-binding modules (CBM), three annotated as cellulases of family GH5, one as a cellulase of family GH12, and another as a cellulase that is homologous to glycosyl hydrolases of GM109 class.

## Discussion

In recent years, the use of renewable resources for different biotechnological applications such as production of second-generation biofuels or other high-added-value products has stimulated research into the discovery of new biocatalysts for efficient conversion of lignocellulosic biomass. An enormous range of different habitats (Amore et al., 2013a) such as soil, compost, decaying plant materials (Zhang et al., 2006; Amore et al., 2013b), rumens (Dai et al., 2012), sewage sludge (Singh et al., 2011), termite guts (Warnecke et al., 2007), forest waste (Ventorino et al., 2013, 2016b), and wood processing plants (Dawkins and Esiobu, 2016), animal feces (Ilmberger et al., 2014), paper mills, and hot springs (López-López et al., 2013) have been so far investigated as sources of both aerobic and anaerobic bacteria producing several different enzymes of industrial interest. In particular, lignocellulosic biomass represents complex ecosystems in which environmental conditions influence living organisms. As a consequence, autochthonous microbial communities with ability to produce enzymes involved in the degradation of (hemi)celluloses may prevail over other microorganisms. Therefore, in this work, 24 Actinobacteria strains previously isolated from the lignocellulosic biomass during natural biodegradation were investigated for their ability to secrete cellulases able to convert the lignocellulosic biomass of *A. donax*.

All the lignocellulosic-adapted strains used in this study were identified as belonging to *Streptomyces* spp. and showed endo-cellulolytic and multiple other enzymatic lignocellulose degrading activities with an attractive potential use in the bioconversion of biomass into fermentable sugars. The majority of the strains were identified as belonging to *S. argenteolus* species demonstrating the high ability of this species to adapt well to lignocellulosic ecosystems. Moreover, the highest cellulase activity production was exhibited by the strain *S. argenteolus* AE58P both in solid and liquid medium. Several works have reported cellulase production in different Actinobacteria, such as *Cellulomonas, Thermobifida*, and *Microbispora*, due to their ability to produce cellulose-hydrolyzing enzymes (Saini et al., 2015). The genus *Streptomyces* has been also previously explored to detect cellulases (endoglucanases, exoglucanases, cellobiases) (Alani et al., 2008; Hsu et al., 2011; Amore et al., 2012), although in the majority of the studies *Streptomyces* have been investigated for their well-known hemicellulase production ability (Beg et al., 2000; Techapun et al., 2003; Raweesri et al., 2008). Recently, one gene from *Streptomyces coelicolor* A3(2), recombinantly expressed in *Streptomyces lividans* TK24, was discovered to encode a protein with strong hydrolyzing activity toward Avicel and filter paper, yielding cellobiose as the final product, and moderate activity toward CMC (~0.4 U mL^−1^) (Lim et al., 2016). El-Naggar et al. (2014) tested the culture supernatant from submerged fermentation of *Streptomyces albogriseolus* subsp. *cellulolyticus* in optimized conditions for the ability to produce endoglucanase and release reducing sugars from agro-industrial residues as substrates. Franco-Cirigliano et al. (2013) screened *Streptomyces misionensis* strain PESB-25 for its ability to secrete cellulases. A peak of endoglucanase accumulation (4.34 U mL^−1^) was observed in optimized fermentation conditions in the presence of MnSO_4_. Amore et al. (2012) identified, recombinantly expressed in *Escherichia coli* and characterized the endocellulase CelStrep from the cellulolytic strain *Streptomyces* sp. G12 isolated from compost. Several strategies have been developed in order to enhance the *Stremptomyces* cellulase production. The integration of the pH-shift and dissolved oxygen (DO)-constant control strategies increased the cellulase and xylanase production from the *Streptomyces griseorubens* JSD-1 strain (Zhang et al., 2016). A response surface central composite design (CCD) was used to optimize the endoglucanase production from the acidothermophilic *Streptomyces* DSK59 strain isolated from soil, adhered to decomposing tree bark (Budihal et al., 2016).

To the best of our knowledge, only two endo-cellulases have been previously detected in a *S. argenteolus* strain (M178) (Tomotsune et al., 2014). The endo-cellulase activity of these proteins was measured after recombinant expression of the respective encoding genes (cel5A_M_ and cel12B_M_) in *Streptomyces lividans*. However, as shown in this work, the highest endo-1,4-ß-glucanase activity level by the native protein(s) from *S. argenteolus* AE58P (0.41 ± 0.05 U mL^−1^) was comparable to that achieved for the recombinantly expressed cel5A_M_ gene by using glucose as unique carbon source (0.409 ± 0.020 U mL^−1^) and higher than that achieved by using CMC as carbon source in the liquid fermentation medium (0.248 ± 0.006 U mL^−1^).

One of the main applications of cellulolytic enzymes and the final goals of their production is the utilization in biomass conversion. Some studies have been carried out in order to test the potential of cellulases from *Streptomyces* strains in the saccharification of different lignocellulose biomasses, such as rice straw (Ball and McCarthy, 1988) and cellulose rich organic materials (Prasad et al., 2012). In the last few years, for sustainable production of biofuels and chemicals, the research interests are focused toward the utilization of crops, such as *A. donax* (common name giant reed), that are not employed for nutritional uses and completely match the principal objectives of the second generation biofuels (Corno et al., 2014). Moreover, this perennial plant was proved to grow in soils not suitable for food crops also reducing soil erosion (Fagnano et al., 2015; Forte et al., 2015) and acting as a phyto-remediating agent (Fiorentino et al., 2010, 2013).

In this scenario, this paper deals with the application of cellulolytic activities for the bioconversion of pretreated giant reed using a cocktail of commercial enzymes and evaluating the potential of the cellulolytic extracts from different *Streptomyces* strains selected among the investigated lignocellulose-adapted ones as the best cellulase activity producers—*S. argenteolus* AE58P, *S. argenteolus* AE710A, *S. argenteolus* AE82P, and *S. argenteolus* AP51A—by substituting them for the commercial counterpart.

When the enzymatic extract from *S. argenteolus* AE58P replaced the Accellerase 1500 preparation (Mix 2), the glucose yield was close to that obtained with the commercial biocatalyst. It is worth noting that the cellulolytic activity from *S. argenteolus* AE58P, the best endo-cellulase producer in solid and liquid medium, also gave the highest glucose recovery in the hydrolysis experiments. Moreover the utilization of the enzymatic extract from this microorganism also resulted in the best xylan deconstruction. Probably the cellulolytic activity from *S. argenteolus* AE58P worked better in combination with the Genencor commercial cocktail than those produced by the other strains. Furthermore, it is possible that a higher glucan deconstruction also had a positive impact on the hydrolysis of xylan, which is strictly absorbed on cellulose fibrils (Dammstrom et al., 2009; Marcolongo et al., 2014). In order to improve the glucose yield, double amounts of each biocatalyst was utilized (Mix 2A, 3A, 4A, and 5A) without achieving better results for glucose yields. It was not surprising since at the higher biocatalyst loadings probably the number of available binding sites on the cellulosic fraction is reduced, hindering a further attack of enzymatic molecules to the substrate (Banerjee et al., 2010). Also the attempt to conduct the biotransformation experiments at a pH value (6.0) more convenient for the commercial biocatalysts did not result in improved glucose yields.

Finally, the obtained results highlighted the applicability of the cellulolytic activities from the strain *S. argenteolus* AE58P in the biotransformation of pretreated *A. donax*. To the best of our knowledge, the ability of only one endo-cellulase from *Streptomyces* strain (named rCelStrep from *Streptomyces* sp. G12, after recombinant expression in *E. coli* and purification) was previously tested in saccharification of pretreated *A. donax* (Marcolongo et al., 2014; Giacobbe et al., 2016). Giacobbe et al. (2016) tested the effect of this endo-cellulase in addition to the commercial preparation Cellic® Ctec3/Htec3 from Novozymes, finding that the glucan and xylan conversion using only commercial enzyme cocktail was higher when compared to that obtained upon supplementation with the purified endo-cellulase rCelstrep. Marcolongo et al. (2014) observed a decrease up to 50% in glucose and xylose yield by using the purified rCelStrep in substitution to Accellerase® 1500 in the same mixture of commercial enzymes from Genencor used in our work. These results were lower than that obtained in the present work by the substitution of the enzymatic extract from *S. argenteolus* AE58P to the Accellerase® 1500. As a matter of fact the yields obtained were very close to those achieved with only the commercial mixture as witnessed by glucose recoveries of 30.17 and 22.05% reached by the utilization of Accellerase 1500 or upon its substitution with *S. argenteolus* AE58P endocellulase, respectively. These data suggested that also in absence of substantial efforts to optimize the process, good conversion efficiencies with the enzymatic extract from the strain *S. argenteolus* AE58P were achieved. Probably better results would be obtained after the removal of the soluble compounds (e.g., sugar, sugar oligomers, sugar degradation products, and lignin-derived compounds) with inhibitory effects on the cellulose digestibility, contained in the slurry of the pretreated biomass. As a matter of fact, higher yields represented by 43 and 70% of glucose and xylose recovery respectively after 72 h of incubation were reached with the same mixture of commercial enzymes from Genencor applied to *A. donax* pretreated by steam explosion but devoid of the liquid fraction containing soluble matter (Marcolongo et al., 2014).

The proteomic strategy applied to the enzymatic crude extracts from *S. argenteolus* AE58P allowed us to identify peptides putatively present in proteins responsible for endo-cellulase activity in this strain. In particular, besides identified oligopeptides showing similarity with glycosyl hydrolases and cellulose-binding proteins from different *Streptomyces* strains, peptides matching peptides present in the only two already known sequences of cellulases from *S. argenteolus* strains were also detected. In particular, five peptides matched to peptides present in the sequence of the GH12 family cellulase (accounting for 20% of its protein sequence) and 4 matched to peptides that are in the sequence of the GH5 family cellulase (accounting for 9% of its protein sequence), both from *S. argenteolus* M178 and previously recombinantly expressed in *S. lividans* (Tomotsune et al., 2014).

## Conclusions

The lignocellulosic-adapted *Streptomyces* strains showed multiple degrading enzymatic activities among which was endo-cellulase activity useful for a potential complete hydrolysis of a cellulose-based complex substrate. Moreover, the kinetics of glucose and xylose release during the saccharification of pretreated *A. donax* biomass by using the endo-cellulase synthetized by the strain *S. argenteolus* AE58P, allowed the glucose yield to reach almost 76% of that obtained in the presence of the commercial counterpart. These results highlighted the biotechnological potential of *S. argenteolus* AE58P being an interesting candidate as a biocatalyst-producing bacterium for lignocellulose conversion and production of biochemicals and bioenergy.

## Author contributions

VV wrote the main manuscript text and, in particular, characterization, and identification of bacterial strains and selection by multienzymatic screening. She prepared Figures 1, 2 and Tables 1, 2. SM wrote the screening of cellulolytic microorganisms in liquid medium and partial characterization of enzymes and prepared Figures 3, 4 and Table 3. FE carried out the identification of bacterial strain. EI and LM wrote the enzymatic hydrolysis of *Arundo donax* and prepared Table 4. LB and AD wrote the identification of endo-cellulase(s) and prepared Table 5. VF and OP conceived the study, participated in its design, and revised the manuscript.

## Funding

This study was supported by grant from the Ministero dell'Università e della Ricerca Scientifica Industrial Research Project “Development of green technologies for production of BIOchemicals and their use in preparation and industrial application of POLImeric materials from agricultural biomasses cultivated in a sustainable way in Campania region – BioPoliS” PON03PE_00107_1/1, funded in the frame of Operative National Programme Research and Competitiveness 2007–2013 D. D. Prot. n. 713/Ric. del 29.10.2010.

### Conflict of interest statement

The authors declare that the research was conducted in the absence of any commercial or financial relationships that could be construed as a potential conflict of interest.
